# Evaluation of the Antioxidant and Antimicrobial Potential of SiO_2_ Modified with Cinnamon Essential Oil (*Cinnamomum Verum*) for Its Use as a Nanofiller in Active Packaging PLA Films

**DOI:** 10.3390/antiox12051090

**Published:** 2023-05-12

**Authors:** Verónica Martínez-Aguilar, Mariana G. Peña-Juárez, Perla C. Carrillo-Sanchez, Leticia López-Zamora, Enrique Delgado-Alvarado, Emmanuel J. Gutierrez-Castañeda, Norma L. Flores-Martínez, Agustín L. Herrera-May, Jose Amir Gonzalez-Calderon

**Affiliations:** 1 Doctorado Institucional en Ingeniería y Ciencia de Materiales, Universidad Autónoma de San Luis Potosí, Sierra Leona No. 550 Col. Lomas 2da. Sección, San Luis Potosí 78210, Mexico; 2 Maestría en Ingeniería y Tecnología de Materiales, Universidad de La Salle Bajío, Av. Universidad 602, Lomas del Campestre, León 37150, Mexico; 3 División de Estudios de Posgrado e Investigación, Tecnológico Nacional de Méxicoen Orizaba, Oriente 9 No. 852 Emiliano Zapata, Orizaba 94320, Mexico; 4 Micro and Nanotechnology Research Center, Universidad Veracruzana, Blvd. Av. Ruiz Cortines No. 455 Fracc. Costa Verde, Boca del Río 94294, Mexico; 5 Facultad de Ciencias Quimicas, Universidad Veracruzana, Blvd. Av. Ruiz Cortines No. 455 Fracc. Costa Verde, Boca del Río 94294, Mexico; 6 Cátedras CONACYT-Instituto de Metalurgia, Universidad Autónoma de San Luis Potosí, Av. Sierra Leona 550 Lomas 2da Sección, San Luis Potosí 78210, Mexico; 7 Ingeniería Agroindustrial, Universidad Politécnica de Guanajuato, Avenida Universidad Sur #1001 Comunidad Juan Alonso, Cortazar 38496, Mexico; 8 Maestría en Ingeniería Aplicada, Facultad de Ingeniería de la Construcción y el Hábitat, Universidad Veracruzana, Boca del Río 94294, Mexico; 9 Cátedras CONACYT-Instituto de Física, Universidad Autónoma de San Luis Potosí, Av. Manuel Nava #64, Zona Universitaria, San Luis Potosí 78290, Mexico

**Keywords:** active packaging, metal oxides, cinnamon essential oil, poly lactic acid

## Abstract

One of the main causes of food spoilage is the lipid oxidation of its components, which generates the loss of nutrients and color, together with the invasion of pathogenic microorganisms. In order to minimize these effects, active packaging has played an important role in preservation in recent years. Therefore, in the present study, an active packaging film was developed using polylactic acid (PLA) and silicon dioxide (SiO_2_) nanoparticles (NPs) (0.1% *w*/*w*) chemically modified with cinnamon essential oil (CEO). For the modification of the NPs, two methods (M1 and M2) were tested, and their effects on the chemical, mechanical, and physical properties of the polymer matrix were evaluated. The results showed that CEO conferred to SiO_2_ NPs had a high percentage of 2,2-diphenyl-l-picrylhydrazyl (DPPH) free radical inhibition (>70%), cell viability (>80%), and strong inhibition to E. coli, at 45 and 11 µg/mL for M1 and M2, respectively, and thermal stability. Films were prepared with these NPs, and characterizations and evaluations on apple storage were performed for 21 days. The results show that the films with pristine SiO_2_ improved tensile strength (28.06 MPa), as well as Young’s modulus (0.368 MPa) since PLA films only presented values of 27.06 MPa and 0.324 MPa, respectively; however, films with modified NPs decreased tensile strength values (26.22 and 25.13 MPa), but increased elongation at break (from 5.05% to 10.32–8.32%). The water solubility decreased from 15% to 6–8% for the films with NPs, as well as the contact angle, from 90.21° to 73° for the M2 film. The water vapor permeability increased for the M2 film, presenting a value of 9.50 × 10^−8^ g Pa^−1^ h^−1^ m^−2^. FTIR analysis indicated that the addition of NPs with and without CEO did not modify the molecular structure of pure PLA; however, DSC analysis indicated that the crystallinity of the films was improved. The packaging prepared with M1 (without Tween 80) showed good results at the end of storage: lower values in color difference (5.59), organic acid degradation (0.042), weight loss (24.24%), and pH (4.02), making CEO-SiO_2_ a good component to produce active packaging.

## 1. Introduction

For decades, polymers have played an important role in our daily lives, contributing to the improvement and implementation of new technologies in countless applications and fields, one of the most important being the packaging industry. The characteristics of a packaging material have evolved over time, adding new functions that allow the protection and preservation of the contained product. Among these new functions is the so-called active packaging, which is characterized by having an active compound capable of inhibiting the growth of microorganisms and other components that favor food preservation. They are generally manufactured with polymers of petrochemical origin due to their good mechanical, thermal, and physical properties; however, as they are formed by very long chains of carbon and oxygen atoms, it is difficult for them to degrade, so there is a tireless search to try to minimize degradation times and environmental problems generated by the use of these polymers [1,2,3].

One of the most promising options is the use of biodegradable polymers for active packagings, such as polysaccharides, cellulose, chitosan, polypeptides, gelatin, zein, polyhydroxyalkanoates, soy protein isolates, and polylactic acid. For the past 30 years, there has been a gradual growth in research focused on the development of active films based on biodegradable polymers [4]. Polylactic acid (PLA) has been used in the development of active films because it is approved by the Food and Drug Administration (FDA) as a safe material (GRAS) for food and beverage packaging [5]. In addition, it has high mechanical properties, tensile strength (45–65 MPa), and particularly Young’s modulus (5–10 GPa), indicating that it is a good substitute for basic polymers such as polypropylene (PP), polyethylene (PL) and polystyrene (PS) in short-life packaging [6,7].

Some research works have already tested the use of PLA in active films, such as Raduzin et al. (2019) [8], who used garlic extract Allium ursinum at 0.5% *w/w,* observing that it improved the crystallinity of pure PLA from 7.3 to 13.2% as well as the Tg from 42.6 to 45.9 °C. Roy et al. (2020) [9] made PLA films with curcumin reporting that at low concentrations (0.25 *w*/*w*%), the tensile strength and Young’s modulus improved from 46 MPa to 49 MPa and 0.88 to 0.94 GPa, respectively, and Fiore et al., (2021) [10] elaborated PLA films with chitosan-caseinate and rosemary essential oil, showing that the mechanical and barrier properties such as water vapor permeability were improved. As we can see in the references presented, active packaging requires two fundamental elements for its manufacture: polymeric matrix and active component. The latter is the key to active packaging since it must have a high antioxidant and antimicrobial capacity.

The most common active components are natural extracts or essential oils, fungicides, bacteriocins, organic acids, and enzymes. Essential oils are obtained from the extraction of plants by distillation; they are very complex mixtures of terpenes (limonene, sabinene, myrcene, valencene, germacrene, etc.), aromatic compounds (ethylbenzene, eugenol, elemicine, apiole, cinnamaldehyde, etc.), and aliphatic compounds responsible for conferring antimicrobial, antifungal, and organoleptic (flavor and aroma) properties [11,12,13]. Each extract has a component of higher proportion that distinguishes it from the others. In citrus essential oil, its main component is limonene (97%); in rosemary, essential oil is 1,8-cineole (45%); in clove, 76.8% is eugenol, oregano essential oil has carvacrol (70%) and cinnamon essential oil has 5% cinnamaldehyde [14,15,16,17,18].

Their antimicrobial activity depends on their polyphenol content. For example, in [19,20,21], it is found that *Cymbopogan citratus* reported a MIC of 0.6 µL/mL for *Escherichia coli*, 0.6 µL/mL for *Staphylococcus aureus* and 2.5 0.6 µL/mL for *Salmonella typhimurium*, while *Psiada argutia* informed a MIC for *S. aureus* of 0.5 mg/mL, of *Enterococcus fecalis* of 8 mg/mL and *Acinetobacter* of 16 mg/mL. Further, *Pimenta dioica* presented a MIC for *E. fecalis* of 2.5 mg/mL, *S. aureus* of 1 mg/mL, and *Acinetobacter* of 8 mg/mL, and finally, *Cinnamomum zeylanicum* reported a MIC value of 2 mg/mL for *Klebsiella pneumoniae*, 4 mg/mL for *E. fecalis* and 0.5 mg/mL for *S. aureus*. In the case of cinnamon essential oil, this reported values of 2.0 mg/mL for *E. coli, S. aureus*, *Salmonella Risen*, and *Pseudomonas fluorescens*, and 0.5 mg/mL for *Bacillus cereus*. Specifically, their inhibition mechanism damages the bacterial cell wall, increasing its permeability due to their lipophilic nature and releasing vital components from inside the cell, leading to cell death. They also apply a reducing action on protein synthesis, intracellular ATP levels, and intracellular pH of bacteria [22,23].

Nowadays, thanks to their properties, essential oils can constitute an alternative to synthetic preservatives and are considered the antibiotics of the future, so we can find in literature several studies of PLA films with essential oils; clove essential oil [24], Oregano essential oil [25], carvacrol and thymol essential oil [26], bergamot, lemongrass, clove and rosemary essential oils [27], cinnamon essential oil [28], Green tea [29] and thyme, rosemary, and oregano essential oils [30]. However, a disadvantage of this semi-crystalline polymer is that its crystallization kinetics is very slow because of the low nucleation capacity. Therefore, this limits its applications since its mechanical properties depend on the degree of crystallinity. Generally, PLA has a crystallization rate in the range of 10 to 20 °C/min at temperatures below 100 °C and a crystallinity of less than 10% due to this poor crystallization ability [31,32]; this is why some material is needed to reinforce and thus improve its crystallinity in order to maintain or even improve its properties.

Nanoparticles of metal oxides are of special interest, and research works have been used for the reinforcement of polymeric matrices, such as titanium dioxide (TiO_2_) [33,34], zinc oxide (ZnO) [35], magnesium oxide (MgO) and (SiO_2_) [36] as they have been used as nanofillers in polymeric matrices. In addition, SiO_2_ nanoparticles are a great option to generate active packaging because, compared to other particles, they are stable, non-toxic, and safe food additives used in food processing and preservation. Negahdari et al. (2020) [35] developed a PLA packaging with Origanum majorana essential oil and ZnO nanoparticles. As a result, they report that the tensile strength decreased by 76% when the essential oil was added, while it increased by 25% when ZnO NPs were added. Water vapor permeability was increased by 3.52 and 1.53 times after the addition of 1.5% essential oil and 1% ZnO NPs, respectively. Their packaging was able to preserve minced meat for 8 days. Alizadeh et al. (2020) [37] performed an active packaging with cellulose nanofibers/serum proteins by adding TiO_2_ nanoparticles and rosemary essential oil and showed that the antioxidant activity was enhanced by adding rosemary essential oil from 42.2 to 47.3 mg/mL which helped the preservation of meat presenting values of 4.15 log CFU/g for the psychrotrophic bacteria count (PBC) keeping the product below the allowed limit (7 log CFU/g). Compared to other metal nanoparticles, silicon oxide (SiO_2_) nanoparticles between 20 and 100 nm have low toxicity, are stable, and are used as FDA-approved food additives [38]. Palma et al. (2021) [36] made PLA films with SiO_2_ nanoparticles (20 nm) at different concentrations (1, 2, 3, 3, 5 wt.%) and found an increase in the percentage of crystallinity from 10 to 77%, as well as the interfacial miscibility was also improved and with it the mechanical properties. Famili Zirak and Tabari (2018) [39] obtained similar results, an increase in the tensile strength from 29 to 43 MPa and Young’s modulus from 2.3 to 3.1 GPa. As can be seen from the above studies, different polymeric matrices, as well as different nanoparticles and essential oils, can be used to produce active packaging.

Considering the above, the present work aims to chemically modify SiO_2_ nanoparticles with cinnamon essential oil (CEO) to improve the antioxidant and antimicrobial properties of these nanoparticles and can function as a nanofiller to confer these properties to the PLA polymeric matrix. The above with the aim of generating an active component in the preparation of films suitable for food preservation. The casting method was used for the preparation of films as active packaging. The effects on the mechanical, thermal, barrier, physical, and microbiological properties of the films were evaluated, and their effectiveness as active packaging was tested by storing a fruit (apple) for a short period of 21 days.

## 2. Materials and Methods

### 2.1. Materials

Silicon dioxide powder SiO_2_ (size 10–20 nm brand IOLITEC Purity 99%) and *Cinnamomum verum* essential oil CEO (brand VidaScent purity 99.99%). Ethanol brand CTR Scientific (purity 99.95% density 0.804 g/cm^3^). Polyethylene glycol sorbitan monooleate Tween 80 (brand Sigma-Aldrich, density 1.07 g/cm^3^, Oleic acid ≥ 58.0%). Commercial polylactic acid (PLA) filament was used to prepare the films. (brand Inky, white, molecular weight 195,000 g/mol, viscosity 109.76 mPa/s to shear rate between 10–100 s^−1^, aspect ratio (L/D) approx. 0.5 with an average length of 2000 ± 0.3 µm, an average diameter of 1750 ± 0.2 µm, and a density of 1.24 g/cm^3^).

### 2.2. Chemical Modification of SiO_2_

Two methodologies were used [40] with minor modifications for this work. On the one hand, the first method (M1) consisted of a mixture of CEO with SiO_2_ in a 1:1 ratio, and 5 mL of ethanol was used for homogenization. The mixture was stirred for 1 h at 30 °C. It is worth mentioning that the CEO is photosensitive; therefore, during the stirring time, the flask was covered with aluminum foil. Finally, the mixture was dried for one day at 50 °C; the modified nanoparticles were labeled as SiO_2_-M1.

On the other hand, the second method (M2) consisted of that 1 g of SiO_2_ dissolved in 5 mL of ethanol by stirring, then 5 μL of distilled water and 2.5 μL of tween 80 were added, and finally, 5 μL of basic cinnamon oil were included. The mixture was left under stirring for 30 min at 30 °C. Then, it was dried under the same conditions that M1, and the modified nanoparticles were labeled as SiO_2_-M2.

### 2.3. Films Preparation of PLA and SiO_2_

**Scheme 1:**
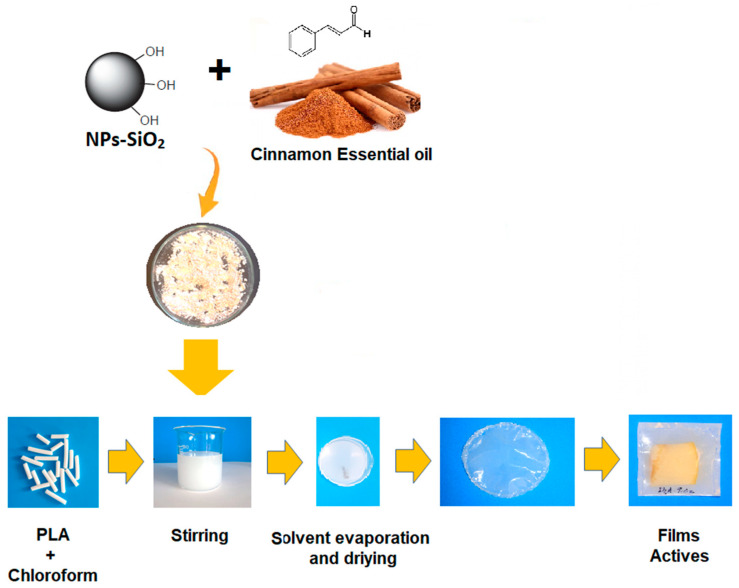
Procedure of the preparation of the PLA films with NPs.

Films of poly(lactic acid) were prepared by the casting method. Initially, 0.500 g of PLA was dissolved in 20 mL of chloroform under stirring. Then, the modified nanoparticles, either by M1 or M2, were incorporated at a rate of 0.1% *w*/*w* regarding the weight of PLA. The mixture was vortexed for 10 min and sonicated for 20 min to eliminate any bubbles and improve the nanoparticles’ dispersion. Finally, the mixture was poured into a petri dish and dried at room temperature. See Scheme 1.

### 2.4. Characterization of the Nanoparticles and Films

#### 2.4.1. Fourier Transform Infrared Spectroscopy (FTIR) and Thermogravimetric Analysis (TGA)

The FTIR spectra of the modified nanoparticles and films were recorded using an Agilent Cary 660 instrument. The analysis was carried out using an Attenuated Total Reflectance (ATR) mode in 4000 to 400 cm^−1^. The thermogravimetric analysis was carried out with the help of differential scanning calorimeter equipment from TA instruments, model SDT 650, with a simultaneous thermogravimetric analyzer. The analysis was conducted with a 10 °C/min ramp in a temperature range of 25–600 °C under a nitrogen atmosphere.

#### 2.4.2. Antioxidant Activity

The antioxidant activity of modified SiO_2_ nanoparticles and films was evaluated using the 2,2-diphenyl-l-picrylhydrazyl (DPPH) free radical method [41]. For the different nanoparticles, several concentrations were tested, which corresponded to each number of moles of antioxidant/mole of DPPH. The prepared nanoparticle solutions were dissolved in ethanol up to final values of 4, 8, 12, 16, and 20 mg/mL. Then, the films were first cut into 5 × 5 cm pieces and dissolved in 2 mL of chloroform. Later, a DPPH solution was prepared with ethanol at a concentration of 6 × 10^−5^ mol/L. An aliquot of 3.9 mL was mixed with 0.1 mL of each nanoparticle solution under stirring. Each solution was incubated in a dark medium at room temperature for 30 min. Finally, the absorbance (abs) of the samples was analyzed in a UV-vis spectrophotometer at 517 nm, and the inhibition % was calculated by Equation (1):

(1) $$Inhibition\;\left(\%\right)=\frac{Abs_{control}-Abs_{sample}}{Abs_{control}}\times 100$$

#### 2.4.3. Antimicrobial Activity

Inspection for microbiological activity is essential in food packaging, as the bacteria present are the cause of spoilage, especially in fruits and vegetables. It is known that *E. coli* can grow in contaminated raw fruits, vegetables, or water. In contrast, S. aureus is a major pathogen found on human skin that may cause infection in several organs during the handling of raw fruits. In order to determine the minimum inhibitory concentration (MIC) of the nanoparticles, the CLSI M7-A7 [42] broth microdilution method was developed. The method consists of inhibiting microbial growth of Gram-positive (*S. aureus*) and Gram-negative (*E. coli*) bacteria in a liquid medium. The first stage consisted of preparing suspensions: nanoparticles were suspended in Mueller-Hinton agar, contained in tubes, and then vortexed for 1 min. The second stage was the preparation of the inoculum, for which *Escherichia coli* and *Staphylococcus aureus* strains were prepared at a concentration of 2.5 × 10^8^ CFU/mL using the McFarlan scale and diluted for a final concentration of microorganisms of 0.5 × 10^3^–2.5 × 10^3^. Microplates (96 wells) were filled with the prepared suspensions and inoculums, considering negative and positive controls. Subsequently, the microplates were incubated with constant stirring at 120 rpm and a temperature of 35 ± 2 °C. Finally, as a third stage, the absorbance of the 96 wells of each system was measured using a Bio-Rad microplate spectrophotometer at 570 nm. Formula (2) was used to determine the growth:

(2) $$\;\%growth=\frac{S-C_{n}}{C_{p}}\times 100$$

where *S* is the absorbance of each sample, $C_{n}$ is the absorbance of the negative control (without inoculum) and $C_{p}$ is the absorbance of the positive control (with inoculum).

A drop test was performed to validate the films’ antimicrobial activity. To start the test, an inoculum of each *E. coli* and *S. aureus* bacteria was prepared as described previously at a concentration of 2.5 × 10^8^ CFU/mL. Then, each film was prepared at a size of 1 cm × 1 cm and was placed for 5 min under UV light to avoid contamination of the medium. Then, each film was placed in sterile 15 mL Falcon tubes, and 3 mL of the inoculated broth was added. They were left to incubate for 24 h at a temperature of 37 °C. In order to determine the percentage of bacterial inhibition, the absorbance was measured with a UV/VIS U-5100 spectrophotometer for each sample using the following Equation (3):

(3) $$\;\%\;inhibition=\frac{A_{0}-A_{m}}{A_{0}}\times 100$$

where *A*_0_ corresponds to the absorbance of the control without sample and *A_m_* corresponds to the absorbance of each sample.

#### 2.4.4. Cell Viability

Cell viability was determined by Resazurin assay using peripheral blood mononuclear cells seeded in 96-well microplates (Costar, Corning, NY, USA) at a cell density of 3 × 10^4^ cells per well in RPMI-1640 medium (10% fetal bovine serum, 2 mM L-glutamine, 50 U mL^−1^ of RPMI-1640 medium). The mononuclear cells were exposed to different concentrations (27.78, 50, 100, and 200 ppm for each system and incubated for 24 h at a temperature of 37 °C in a controlled atmosphere (5% CO_2_ and 95% relative humidity). Before reading the samples by spectrofluorometry, the cells were treated with Resazurin (Sigma-Aldrich, St. Louis, MO, USA) at 30 µg mL^−1^ diluted in RPMI medium in a final volume of 100 µL per well. Microwells were immediately placed in an incubator at 37 °C and 5% CO_2_ atmosphere and protected from light. Fluorescence measurements were determined at emission wavelength λ = 590 nm and excitation wavelength λ = 560 nm. Samples were measured with a Synergy H1 spectrofluorimeter (BioTek Instruments Inc. Winooski, VT, USA). Untreated peripheral mononuclear cells were used as the negative control, and cells treated with acetone (10%) (Sigma-Aldrich, St. Louis, MO, USA) were considered the positive control. Cell viability of all systems was determined in triplicate with Equation (4):

(4) $$Cell\;viability\;\left(\%\right)=\frac{\;f_{s}}{f_{c}}\times 100$$

where *Fs* is the fluorescence value of each sample and *Fc* is the fluorescence of the control.

#### 2.4.5. X-ray Diffraction (XRD) and Scanning Electron Microscopy (SEM)

XRD diffraction spectrograms were obtained using an X-ray diffractometer (D8 ADVANCE, Bruker) using a Cu Kα radiation beam filtered with nickel, with an angular range of 8–80° at a step of 2θ and voltage of 40 kV. Samples were cut into small frames of 1 × 1 cm. The prepared films were analyzed with a Philips/FEI XL30-SFEG high-resolution microscope. A 2 × 2 cm sample was cut out, placed on pins, and coated with a thin layer of silver from the central part of the films. The inspection was made on the surface of each film at an accelerating voltage of 20 kV.

### 2.5. Physical Properties of the Films

The samples were conditioned 24 h before each measurement in Section 2.5 at a relative humidity of 55% and a temperature of 25 °C.

#### 2.5.1. Film Thickness (FS)

A high-precision (0.001 mm) digital micrometer (ULINE H-7352) was used to determine the thickness of the films. Measurements were taken at 5 different points in each film. The average value of the film thickness was used to determine the mechanical and physical properties of the film with the modified SiO_2_ nanoparticles.

#### 2.5.2. Water Solubility (WS)

Water solubility can be described as the solubilized dry matter content after 24 h immersion in water. The method for determining water solubility is described as follows: in a laboratory oven, the samples were dried for 24 h at 50 °C until constant weight and then weighed (*W*1). The samples were then immersed in 40 mL of distilled water for 24 h at room temperature under magnetic stirring. After this time, the film was removed and dried at 110 °C for 24 h until constant weight and was weighed to determine the weight of the final matter (*W*2) that had not dissolved in water [43]. Formula (5) was used to determine the percentage solubility in water.

(5) $$\;\%WS=\frac{\left(W1-W2\right)}{W1}\times 100$$

#### 2.5.3. Water Vapor Permeability (WVP)

A gravimetric method of ASTM E-96-95 [44,45,46] was chosen to analyze water vapor permeability. First, a solution of 142 mmol/L of sodium ions was prepared with NaCl and mixed with a solution of 2.5 mmol/L of calcium ions, prepared previously with CaCl_2_. Then, an aliquot of 10 mL of this mixture was taken and poured on a petri dish 5.4 cm in diameter. Finally, a film was placed on the petri dish and adjusted with a rubber band to prevent the film from moving. The weight of the Petri dishes containing the film and the solution was recorded. They were placed in a desiccator containing silica gel.

The desiccator was placed in a temperature-controlled chamber for 6 h at 34 °C (according to the described in the ASTM standard E-96,88), where the weight of each box was recorded every hour. To know the water vapor permeability, Formulas (6) and (7) were used, expressing the water vapor transmission rate as *WVTR*, which is the water vapor transmission rate Δ*m*/Δ*t* in g and h, respectively; *WVP* is the water vapor permeability, *A* is the exposed area of the film in m^2^, *X* is the film thickness in m^2^, *P* is the saturation vapor pressure of water in Pa at the test temperature (25 °C) finally, R1 is the relative humidity in the desiccator which was 40% and R2 the relative humidity inside the Petri dish.

(6) $$\;WVTR=\frac{\Delta m}{\Delta t\cdot A}\;$$

(7) $$WVP=\frac{WVTR}{P\left(R2-R1\right)}X$$

#### 2.5.4. Contact Angle (CA)

Measurements of water contact angles were performed by observing the intermolecular interactions between the surface of the films and a small droplet (1 µm) placed with a micropipette. Several images were taken through a camera. The contact angles were measured using ImageJ software version 1.54d with the drop analysis function.

#### 2.5.5. Mechanical Properties

In order to estimate the mechanical properties of each film, the method described in ASTM D882-18 [47] was followed with some modifications. The tests of the tensile strength (TS), elongation at break (EB), and Young’s modulus (YM) were evaluated. The samples were customized in 1 × 9 cm films, and the analytical measurements were performed in quintuplicate in a texture analyzer (Texture Pro CT Build 35) under the following conditions: the initial grip of 15 mm and a crosshead speed of 1 mm/s. Tensile strength (MPa), elongation at break (%), and Young’s modulus (MPa) were calculated using the formulas described in Equations (8)–(10), respectively.

(8) $$\;TS=\frac{F_{max}}{A}$$

(9) $$\%EB=\frac{L}{L_{0}}\times 100\;$$

(10) $$YM=\frac{F\;L}{\Delta L\;A}$$

where *F_max_* is the force at break (N), *A* is the cross-sectional film area (mm^2^), *L*_0_ is the initial film length (mm), and *L* is the film length at break (mm). Young’s modulus was estimated as the slope of the elastic region in the stress-strain curve. Strain (ε) = (L − L_0_)/L, stress (σ) = F/A (MPa).

#### 2.5.6. Differential Scanning Calorimetry (DSC)

The thermograms on films were obtained by a Perkin Elmer model 8500 Differential Scanning Calorimeter. The sample was placed in a small, hermetically sealed aluminum capsule containing between 6 and 15 mg. The samples were scanned at 10 °C/min over a temperature range of 25–200 °C. The Tg (glass transition temperature), Tc (crystallization temperature), and Tm (melting temperature) were obtained. The Equation degree of crystallinity (Xc) of the samples was calculated according to the following Equation (11):

(11) $$\;Xc\left(\%\right)=\left(\frac{\Delta H_{m}-\Delta H_{c}}{\Delta H{{}^{\circ}}_{m}}\right)\times 100$$

where $\Delta H_{m}$ is the enthalpy of fusion, $\Delta H_{c}$ enthalpy of crystallization and $\Delta H{{}^{\circ}}_{m}$ (93 J/g) is the heat of fusion for 100% crystallinity of PLA homopolymer [48].

### 2.6. Storage and Evaluation of Active Packaging in Apple Samples

Golden Delicious apples from Saltillo Coahuila were obtained from a local grocery store. The apples used during the storage test were 2 × 3 cm in size per piece. These apples were packed with properly processed and heat-sealed film. The storage time of the apples was 21 days at 15 °C; in addition, complementary tests of color, pH, acidity, and weight loss were performed weekly to evaluate the behavior of the fruit stored and packed with this active package.

#### 2.6.1. Weight loss

The weight loss, expressed as a percentage, was determined as follows (12):

(12) $$Loss\;weigth=\left[\frac{W_{0}-W_{t}}{W_{0}}\right]\times 100\;$$

where *W_t_* is the weight of the apple sample after each week, *W*_0_ is the initial weight of the sample.

#### 2.6.2. Total Titratable Acidity (TA)

Titratable acidity (TA) in apple samples was determined following the method described by Velickova et al. [49]. The method consisted of crushing 5 g of apple in a mortar and pestle, adding 50 mL of distilled water, then increasing the room temperature to 80 °C for 30 min without stirring, and finally, filtering. NaOH 0.1 mol/L was then used to titrate the solution to a pH of 8.1. The formula described (13) was used to determine the percentage of titratable acidity.

(13) $$\;TA=\left[\frac{V_{\left(NaOH\right)}\times 0.1\times 0.064}{m}\right]\times 100\;$$

where *V*_(NaOH)_ is the volume (mL) of NaOH added, 0.1 is the molarity of the NaOH solution, 0.064 is the conversion factor for malic acid, and *m* is the mass of the sample.

#### 2.6.3. pH Evaluation

A Hanna Instrument potentiometer was used to determine the pH of the apple samples, which were previously crushed with a mortar and pestle. Then, 50 mL of distilled water was added. In order to avoid pulp residues, the solution was filtered, and the measurement was performed once the equipment was calibrated.

#### 2.6.4. Color Evaluation

The colorimetric test to determine the color of the packaged apple piece was carried out in two stages, the first before packaging (initial value) and the second after 21 days (final value). A color Muse SE pocket colorimeter was used for this test. The main parameters of the Hunter Lab color space, L* (lightness), a* (position between red and green), b* (position between yellow and blue), and the color difference (∆E) obtained by Equation (14) were considered:

(14) $$\Delta E=\sqrt{{\left(L_{0}-L_{f}\right)}^{2}+{\left(a_{0}-a_{f}\right)}^{2}{\left(b_{0}-b_{f}\right)}^{2}}\;$$

where subindex 0 is the initial value and *f* is the final value.

### 2.7. Statistical Analysis

An analysis of variance (ANOVA) was used to determine the difference in means of the data obtained, using the Minitab software package for Windows (19.1.1.0) using Tukey multiple range test analysis with a significance level of *p* < 0.05.

## 3. Results

### 3.1. Characterization of Nanoparticles

#### 3.1.1. Fourier Transform Infrared Spectroscopy (FTIR)

**Figure 1:**
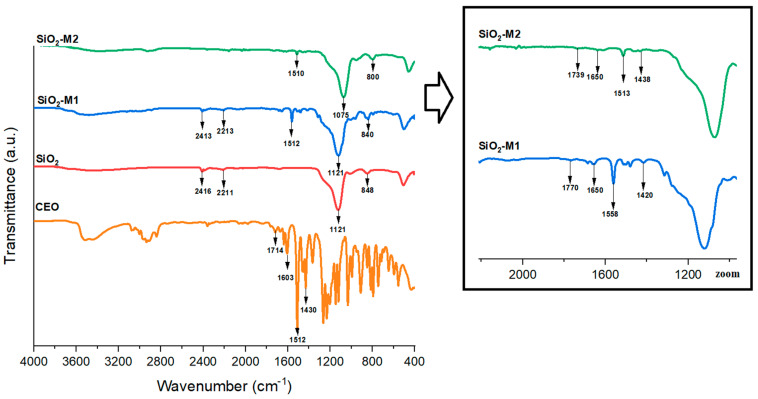
FTIR spectrum of nanoparticles SiO_2_ pure, SiO_2_-M1, SiO_2_-M2 and CEO.

The Fourier transform infrared spectrum provides the necessary information to identify functional groups. With the above in mind, pure and modified SiO_2_ nanoparticles (M1 and M2) were analyzed to verify that the main compound (cinnamaldehyde, 81.2%) of cinnamon essential oil (CEO) [50] was bonded on SiO_2_ particles. Firstly, it can be appreciated in Figure 1 the absorption bands for pure SiO_2_, which appeared at 848 and 1121 cm^−1^ and are attributed to the symmetric and asymmetric stretching of (Si-O), respectively. The bands at 2416 and 2211 cm^−1^ correspond to the (-OH) stretching of the hydroxyl groups present on the surface of SiO_2_ nanoparticles. Previous studies indicate [31,32] that the characteristic bands of SiO_2_ are found at 1105 cm^−1^ due to the symmetric (Si-O-Si) stretching and bending mode. The (Si-O) and (O-Si-O) stretching vibrations can be observed between 803 and 450 cm^−1^. These bands have been observed by Marangoni et al. [33] and Hernández et al. [34].

Figure 1 also illustrates the FTIR spectrum of CEO, which showed the absorption bands at 2938, 2834, 1709, 1603,1505 1503, 1232, 1157, 1031, and 904 cm^−1^. Several studies mention that the characteristic peaks of CEO are found at 2827–2884 cm^−1^ and correspond to the (C-H) stretching, while at 1687 cm^−1^ is found the aldehyde (C=O) stretching, at 1643 cm^−1^ the alkene (C=C) stretching, and at 1423 cm^−1^ refers to the aromatic (C-C) ring [50]. In contrast, the bands found in the systems (SiO_2_-M1 and SiO_2_-M2) prepared by methods M1 and M2 are located at 1075, 1616, 1510, 1438, 800, and 1770, 1650, 1558, and 1420 cm^−1^ respectively. It is important to mention that the bands of system M1 were more intense; this could be attributed to a large dipole moment between molecules. In other words, there was a stronger interaction between CEO and SiO_2_ nanoparticles (SiO_2_-M1) compared to method two (SiO_2_-M2) when tween 80 was added, which could be interpreted as the presence of this compound prevents the direct binding of the functional groups of CEO on SiO_2_ nanoparticles. Compared to the spectrum of pure SiO_2_, it can be seen that there is no presence of these bands, indicating that the characteristic cinnamaldehyde group of CEO is present only on the modified SiO_2_ nanoparticles.

#### 3.1.2. Thermal Analysis (TGA)

**Figure 2:**
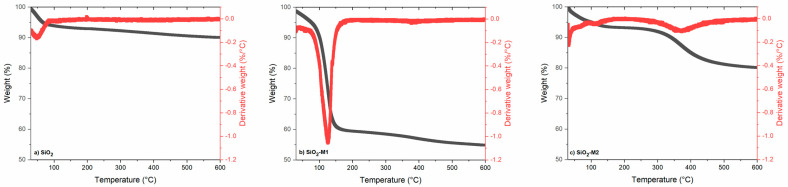
TGA of the nanoparticles (**a**) SiO_2_ pure, (**b**) SiO_2_-M1 and (**c**) SiO_2_-M2.

The TGA results are shown in Figure 2, where the thermogram of pure SiO_2_ nanoparticles can be seen, which remains without weight loss during a temperature range from 0 to 600 °C due to the nature of this compound, i.e., because SiO_2_ is a pure compound, no curves referring to the loss of other elements were observed; therefore, its graph remains constant. The thermal decomposition of the nanoparticles modified with method two can be observed as the mass loss in two stages, firstly the evaporation of the CEO essential oil occurs with a value of 10%, and in the second stage, there is a loss of 20% when a temperature of 350 °C is reached corresponding to the degradation of Tween 80. Similar results were observed by Cao and Song [51]; Liu et al. [52] and Zhou et al. [53] mention that in this temperature range of 130–369 °C the weight loss in this stage is related to the decomposition of Tween 80 and the degradation of other components. In contrast, it is observed that the nanoparticles of method one possess a higher mass loss of about 40% at a temperature of 50–150 °C due to the deterioration of the volatile/non-volatile components largely present in a cinnamon essential oil [54]. The results of nanoparticles modified with both methodologies demonstrate that the interaction of SiO_2_ with CEO improves the thermal stability of the oil [55]. Similar results were obtained by Wen et al. [56] when evaluating the thermal stability in the encapsulation of cinnamon essential oil in nanofibrous films for active food packaging, observing that the degradation of CEO occurs at approximately 100 °C, also mentioning that the thermal stability in the PVA nanofibrous film with CEO was improved. Likewise, Zhou et al. [53] evaluated the effect of CEO addition on edible cassava starch films. This gives us an indication that the thermal stability will be maintained in a wide range, and therefore, these nanoparticles can be used in packaging due to this stability and the relationship between the cinnamon essential oil components within the polymeric matrix [57].

#### 3.1.3. Evaluation of Antioxidant Activity of Nanoparticles

**Figure 3:**
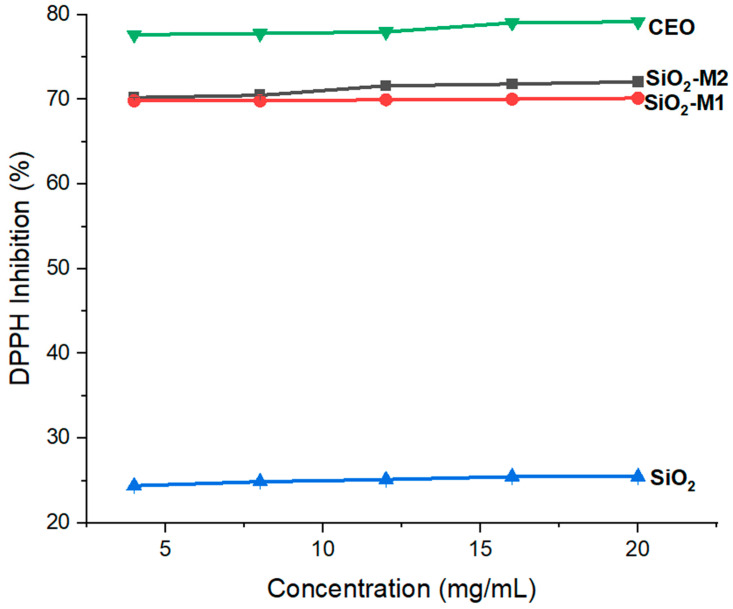
Antioxidant capacity of nanoparticles SiO_2_ pure, SiO_2_-M1 and SiO_2_-M2.

Once it was confirmed that the CEO was present in the SiO_2_ nanoparticles, its antioxidant power was determined in the nanoparticles modified by both methods (M1 and M2) to know which maintained a higher percentage of free radical inhibition. Figure 3 shows the results obtained for DPPH free radical inhibition. The pure CEO presents a high percentage of inhibition between 77–79%, while the SiO_2_-M1 and SiO_2_-M2 modified nanoparticles present values between 69–70% and 70–72%, respectively. These results indicate that the nanoparticles acquired the antioxidant power of CEO at 88% and 91%, respectively. In contrast, it can be observed in Figure 3 that pure SiO_2_ nanoparticles alone do not present a high antioxidant capacity, reaching only 24–25% inhibition. In this context, it is possible to establish that the phenolic compounds in cinnamaldehyde help inhibit the release of radicals, upon reacting with oxygen, accelerate their biological process, causing autooxidation [58,59]. It is worth mentioning that the percentage of inhibition was measured at different concentrations, so it is observed that the antioxidant capacity depends only on the functional groups present in the particles; This is relevant because, at a minimum concentration (4 mg/mL), the same percentage of inhibition is achieved as at a higher concentration (4 mg/mL). Studies such as that of Guo et al. [60] reported a percentage inhibition of pure CEO between 88.18 and 59.35% using various cinnamon essential oil extraction methods. So far, few previous studies have evaluated CEO antioxidant power in metal oxide nanoparticles. There is only one previous work as a reference [50] where an inhibition percentage of 60% was reported in TiO_2_ nanoparticles (size 500 nm) lower than that obtained in this study, concluding that the size of the nanoparticles used is an important factor for chemical modification with essential oils.

#### 3.1.4. Microbiological Tests

**Figure 4:**
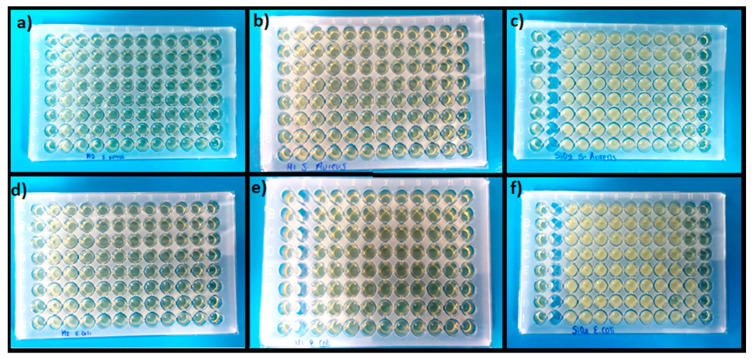
Microplates for antimicrobial evaluation for *S. aureus*: (**a**) SiO_2_-M2 (**b**) SiO_2_-M1 and (**c**) SiO_2_ pure; *E. coli* (**d**) SiO_2_-M2 (**e**) SiO_2_-M1 and (**f**) SiO_2_ pure.

Another property of interest for the manufacture of active packaging is the antimicrobial activity of the active component, so the objective of this assay was to evaluate the minimum inhibitory concentration and its effect on Gram-negative and Gram-positive bacteria. In this context, the antimicrobial capacity of SiO_2_ nanoparticles modified with the M1 and M2 methods was evaluated against two types of E. coli and S. aureus bacteria using the microdilution technique at different concentrations. In summary, Table 1 shows the results obtained for the minimum inhibitory concentration (MIC) of each system. It is possible to observe in Figure 4c,f that pure SiO_2_ does not present any antimicrobial capacity since it did not inhibit the growth of bacteria, which is observed as turbidity to the naked eye. In contrast, the wells of the other sections are clear and translucent, which is an indication of microbial inhibition. In contrast, inhibition was observed for the SiO_2_-M1 system, with a MIC of 45 µg mL^−1^, while for the SiO_2_-M2 system, it was 11 µg mL^−1^ for *E. coli*. This indicates that the CEO conferred antimicrobial properties to the nanoparticles by inhibiting the growth of Gram-negative bacteria. This is due to the monoterpenes present in the CEO, which are responsible for generating pH alterations in the cell membrane, as well as increasing the permeability of the membrane and causing proteins and nutrients essential for the cells to escape until they die [61]. As for *S. aureus* bacteria, the MIC for systems M1 and M2 was 90 and 750 µg mL^−1^, respectively. A higher concentration of nanoparticles was necessary to inhibit growth against this Gram-positive bacterium. The inhibitory capacity of Gram-positive bacteria could be due to the fact that they do not have an outer membrane around the cell wall, which facilitates the diffusion of hydrophobic compounds from the essential oils [62,63]. Some research works, such as Nemattalab et al. [64], mentioned that by fabricating solid lipid nanoparticles loaded with CEO, they obtained a MIC of 65 µg mL^−1^ for *E. coli* bacteria compared to nanoparticles without CEO. Jabir et al. [65] fabricated gold nanoparticles modified with glutathione and linalool and reported a MIC for *E. coli* bacteria of 0.77 µg mL^−1^ and 1.54 µg mL^−1^ for *S. aureus*. Elghobashy et al. [66] elaborated on chitosan nanocomposites with alginate loaded with thyme and essential garlic oil and showed that the bactericidal efficacy of essential oils improved when these were combined, the MIC obtained for *E. coli* was 15 µg mL^−1^, while for *S. aureus* it was 7.5 µg mL^−1^, and these values were lower than those obtained. We can say that essential oils are effective against the inhibition of Gram-positive and Gram-negative bacteria when transferred to other materials.

#### 3.1.5. Cell Viability Test

**Figure 5:**
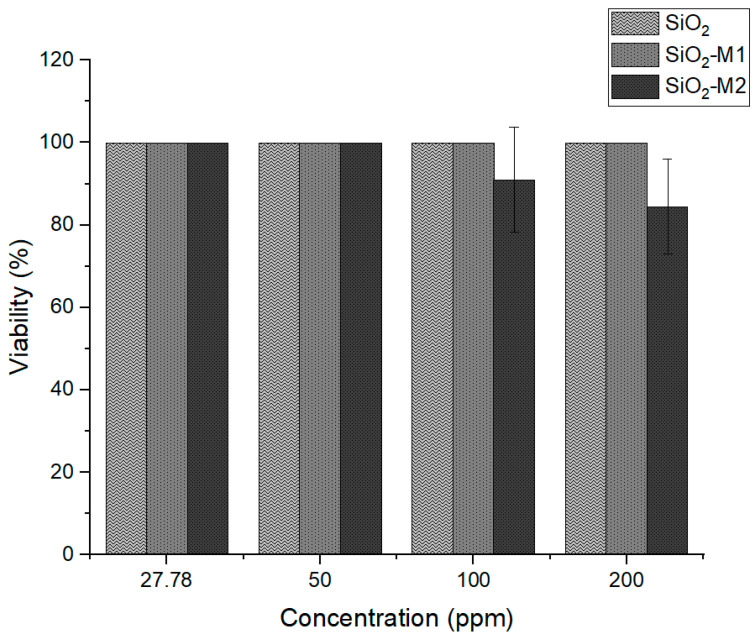
Percentage of cell viability of the nanoparticles SiO_2_ pure, SiO_2_-M1, and SiO_2_-M2.

Once the antioxidant and antimicrobial activity of SiO_2_ nanoparticles modified with essential oil was corroborated, it was necessary to know the suitability of these nanoparticles for their application as active packaging. As they are intended for the food sector, they should not generate a health risk, so their toxicity level was quantified using the cell viability assay. The study results are shown in Figure 5 at an exposure time of 24 h; the nanoparticles treated with method one (SiO_2_-M1) and pure SiO_2_ presented 100% cell viability at different concentrations on average. In contrast, nanoparticles treated with method two (SiO_2_-M2) showed a decrease in cell viability with increasing concentration; however, cell viability remained above the 80% allowed, and according to ISO 10993-5:2009, the material has low toxicity. This may be because the nanoparticles have been treated with Tween 80, unlike methodology 1, so it could be assumed that it is the Tween 80 that causes this effect and not the interaction of the cinnamon essential oil. As expected, the toxicity is low, giving a guideline to develop active packaging for food applications. Similar results have been obtained by Al-Tayyar et al. [67] showing low toxicity in mixtures of PVA nanocomposites with chitosan and ZnO-doped SiO_2_ particles.

### 3.2. Characterization of the Films

#### 3.2.1. FTIR

**Figure 6:**
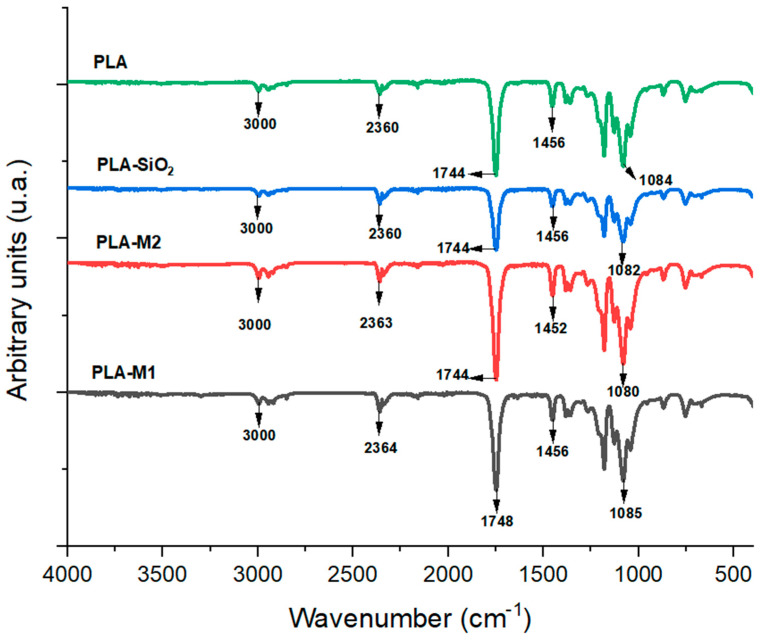
FTIR spectrum of films PLA pure, PLA-SiO_2_, PLA-M1, and PLA-M2.

In order to evaluate the modifications in the structure of the PLA polymeric matrix, infrared spectroscopy analysis was performed. Figure 6 illustrates the FTIR spectrum for PLA films and films reinforced with pure SiO_2_ particles and those modified with the M1 and M2 methods. Absorption signals can be observed at wavelengths of 867 cm^−1^ and 754 cm^−1^, corresponding to the PLA amorphous phase and crystalline phase, respectively [68]. Bands also appeared at 1183 and 1081 cm^−1^, which are attributed to the symmetric and asymmetric stretching of the ether group (C-O-C) [46]. The bands appearing at 1453 and 1358 cm^−1^ correspond to saturated hydrocarbons’ asymmetric and symmetric stretching vibration (CH_3_-) [69]. The band at 1744 cm^−1^ is attributed to the stretching vibration (C=O) of the ester group of PLA molecules [70]. The peaks observed at 2933 cm^−1^ correspond to the CH_3_- stretching vibrations, and the bands observed at 1628 and 1506 cm^−1^ are assigned to the carbonyl and ethylene groups, respectively [71]. In the film samples, no significant changes were observed in the spectra obtained; only slight changes in peak intensities were observed. This indicates that no noticeable changes occurred in the chemical structure of PLA since no new chemical bonds were formed between PLA and pure and modified SiO_2_ particles. These results were also observed by Lukic et al. [72] and Mohamad et al. [73] on PLA films with essential oils in the polymer matrix, showing that there are no significant chemical changes in the films after the addition of active agents.

#### 3.2.2. XRD

**Figure 7:**
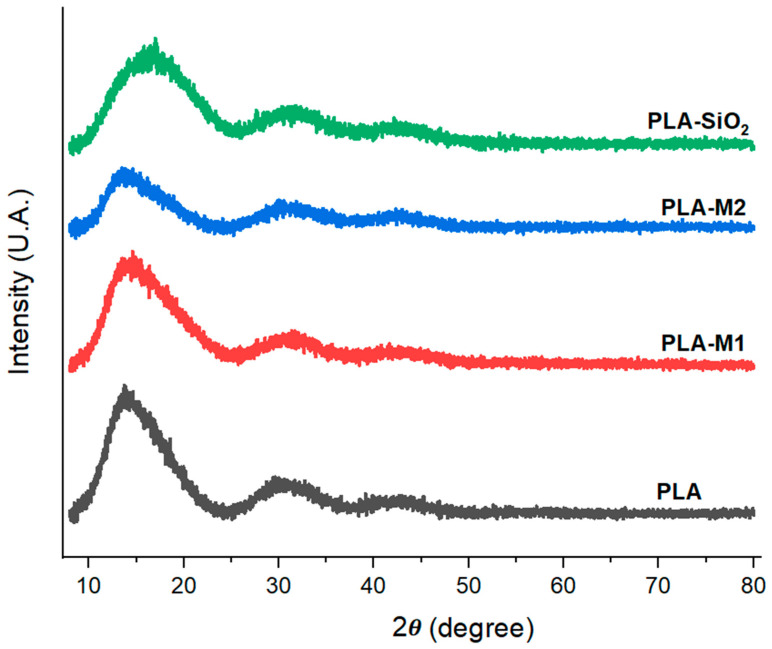
XRD pattern of films PLA pure, PLA-SiO_2_, PLA-M1, and PLA-M2.

In order to know the effects of SiO_2_ with essential oil on the films, the diffractograms were obtained. In Figure 7, where it can be observed that all the films prepared with both pure SiO_2_ and modified nanoparticles presented similar characteristics. A broad peak of large protrusion with an angle of 2θ = 16.89° is attributed to the semi-crystalline nature of PLA and corresponds to the reflection of the α (110/200) crystalline shape plane [72]. The film formulated with the M1 method showed a slight variation, which can be quantified by calculating the percentage of crystallinity, which was 52.56. At the same time, the pure PLA had a value of 55.01%.

On the one hand, it was observed that when films were prepared with nanoparticles from the M1 method, the PLA crystallinity was reduced by 4.45%. On the other hand, in the film with nanoparticles modified with method 2, it is observed that this same peak decreases its intensity considerably, indicating that this interaction is increasing the disorder of the polymeric chains by 31% since its percentage of crystallinity was calculated at 37.91%. In contrast, the films formulated with SiO_2_ show a significant change in curvature due to the presence of these nanoparticles in the matrix, generating a slight broadening of the peak found at 16.89°, in addition to the fact that their crystallinity percentage was 49.97%. This could be because SiO_2_ is in an amorphous state, which generates, as in the previous case, the disorder of the polymeric chains. It was demonstrated that the effect caused by the addition of CEO in the PLA matrix reduces the crystallinity. This same effect was observed in other investigations. Valenzuela et al. [74] reported that the introduction of sunflower oil in a quinoa protein and chitosan-based film generated a less crystalline structure. Likewise, Ahmed et al. [75] indicated that there were no significant variations in the structure of PLA films when formulated with other materials, such as graphene oxide nanofillers.

#### 3.2.3. SEM

**Figure 8:**
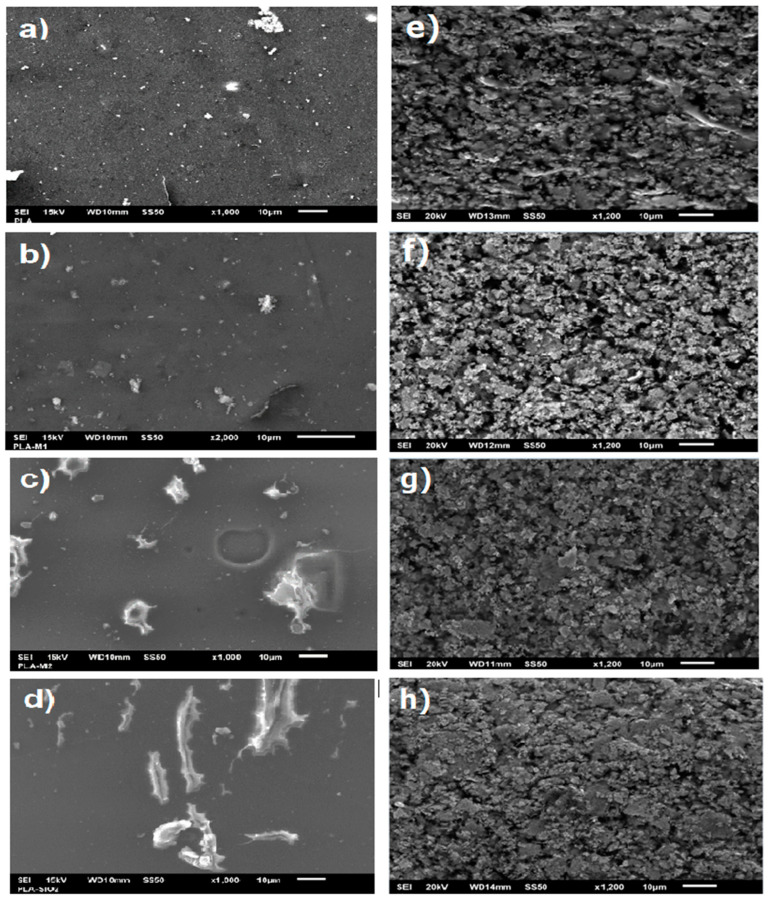
SEM micrographs of films: (**a**–**d**) surface and (**e**–**h**) cross-section of (**a**) PLA, (**b**) PLA-M1, (**c**) PLA-M2, and (**d**) PLA-SiO_2_.

A scanning electron microscopy (SEM) analysis was performed to evaluate the effects on the morphology of the films. Figure 8 shows the SEM micrographs of the films prepared from pure PLA, PLA with pure SiO_2_, and nanoparticles modified with the essential oils (M1 and M2). In all the films, some areas that could correspond to TiO_2_ nanoparticles are observed since PLA is fabricated with rutile as a white pigment. In the case of pure PLA films, only these TiO_2_ nanoparticles are observed; on the contrary, it can be observed that the film containing pure SiO_2_ nanoparticles did not develop a good interaction of the nanoparticles with the polymeric matrix since agglomerations and non-homogeneous distribution are appreciated. See Figure 8.

Similarly, the film with M2 nanoparticles showed small agglomerations, and some scratches, possibly due to the Petri dishes marks; however, there is a better distribution of the nanoparticles compared to pure SiO_2_. Likewise, it can be observed that the film with M1 nanoparticles has a more homogeneous distribution, is smoother, and does not present scratches or cuts. In the same way, we can observe in the sections (e–h) the cross sections of the films, where a rough texture with holes is observed; in the PLA-M1 film, better dispersion of the nanoparticles is observed, for PLA-M2, a uniform distribution is observed in the same way, but with some agglomerations, while the PLA-SiO_2_ film shows larger agglomerations that generate rougher films, we can say that the film that had better miscibility with PLA was the one containing nanoparticles modified by method 1. This is the case presented by Mahmood et al. [76] and Yang et al. [77], who developed films with TiO_2_ as a nanofiller and obtained a heterogeneous dispersion with lumps and rougher fractured surface than the PLA film. On the other hand, the study by Lukic et al. [72] mentions that the interaction of PLA with essential oils without nanofillers generates less smooth films and the formation of large voids on the surface of the films. Given the above, it indicates that the proposed methods improve the morphology of the films.

#### 3.2.4. Antioxidant Activity in Films

**Figure 9:**
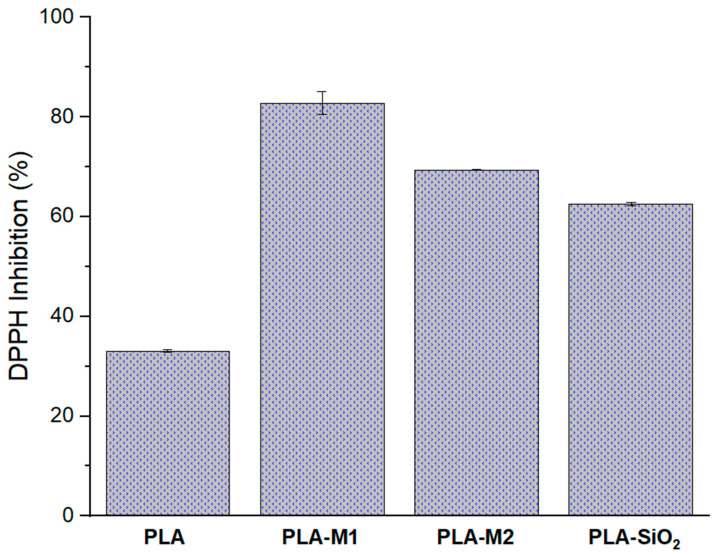
Antioxidant capacity of PLA films, neat and filled with M1, M2, and SiO_2_.

Another important characteristic to evaluate in active packaging is its antioxidant activity in the film. This property allows the stored product to be preserved for longer, maintaining its nutritional properties and appearance since the consumer’s acceptance or rejection of a product depends on this. The results obtained are shown in Figure 9 it can be observed that the films prepared with SiO_2_ nanoparticles presented a higher percentage of free radical inhibition compared to pure PLA; these values were 82.81%, 69.38%, and 62.55% for the PLA-M1, PLA-M2, and PLA-SiO_2_ systems, respectively. When these values are compared with those measured for nanoparticles modified with essential oil, it can be observed that this property increased by 15% for the PLA-M1 system, in contrast with the PLA-M2 system, which decreased by approximately 6.94%. Something interesting that can be observed is that the PLA-SiO_2_ system increased its inhibition percentage by 60% compared to that obtained by the nanoparticles; this could be because the PLA used in white color and has TiO_2_ in its composition which gives it that coloration, so it is assumed that TiO_2_ is helping to inhibit free radicals, that is why these increases were observed, Alizadeh et al. [37] reported that at a minimum concentration of 25 mg/mL TiO_2_ pure is able to inhibit 20% of DPPH. Likewise, Martínez et al. [40] found that at a concentration of 4 mg/mL, TiO_2_ had a 20% inhibition. Alam et al. [78] reported that at 20 µg/mL, TiO_2_ presented 14% inhibition to DPPH. Several studies, such as Lukic et al. [72], Kumar et al. [79], and Mariño et al. [80], mention that there are high antioxidant activity values in films containing essential oils, making these active components ideal for use in active packaging.

#### 3.2.5. Microbiological Analysis of Films

After confirming the antimicrobial activity of the nanoparticles modified with essential oil, a microbiological test was performed on the films to determine if this property could also be transferred to the film, which is an important property in active packaging. In Table 2 it can be observed the drop test results performed on the films. The pure PLA system had practically no inhibition, with values of 0.43% and 0.92% for both *E. coli* and *S. aureus* bacteria. In comparison, the systems prepared with nanoparticles modified with the essential oil presented mean values between 1.29 and 3.54% for Gram-positive and Gram-negative bacteria. Such low values could be because the diffusion of the nanoparticles is not so fast, which prevents higher antimicrobial activity in the films [81,82,83,84]. However, this result also indicates that the nanoparticles have well adhered within the polymeric matrix, so there is no migration, which could favor the properties of the active packaging as the slow diffusion could help preserve food for a longer time. Similar to the antimicrobial activity of the nanoparticles, the films followed a behavior of higher inhibition of E. coli bacteria due to the thicker cell wall of S. aureus, which generates a higher force needed to inhibit the bacteria. As can be observed, the PLA-SiO_2_ system did not have a significant difference between both bacteria, having a very similar value to pure PLA; this could be attributed to the fact that pure SiO_2_ does not have any inhibitory effect against these bacteria according to what it could be obtained in the microbiological analysis of the nanoparticles. The study by Montero et al. [85] on cellulose nanofiber films loaded with CEO observed the same behavior using the disk diffusion technique, where the films did not show inhibition halos; however, the antimicrobial effect is more visualized when packaging a product inside, they stored strawberries which were kept in good conditions for 15 days.

### 3.3. Evaluation of Physical Parameters in Films

#### 3.3.1. Water Solubility (WS)

**Figure 10:**
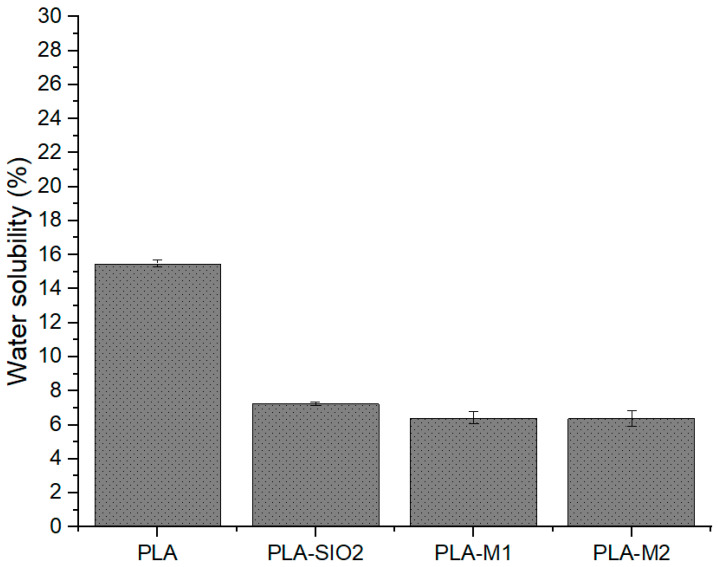
Percentage solubility in water of films PLA pure, PLA-SiO_2_, PLA-M1, and PLA-M2.

An important factor when choosing a biopolymer is its solubility in water since knowing this parameter can guide the application to food product packaging [86,87,88]. The results obtained are illustrated in Figure 10 and Table 3, which show a significant variation in the solubility of pure PLA of 15% concerning films formulated with SiO_2_ nanoparticles and those modified with cinnamon essential oils. The films with modified particles maintained low solubility percentages, between 6% and 7%, which indicates that the films are resistant to degradation in aqueous media, making them suitable for food storage at low temperatures with higher percentages of relative humidity. This could be due to the hydrophobic interaction of the essential oils in the polymeric matrix. Previous studies mention that this physical property can be affected positively or negatively, depending on the interaction between the components, for example, increased solubility percentages in studies such as that of Cai et al. [89]. Similar results obtained by Hadi et al. [87] and Cao and Song et al. [51] showed a reduction in water solubility with increasing lemon essential oil (EO) concentration, ranging from 46.2% (control) to 38.7% and 33.4% with the addition of 1% and 2% EO. These results are possibly due to the hydrophobic character of the essential oil in the films and the interaction with the hydroxyl groups of water.

#### 3.3.2. Water Vapor Permeability (WVP)

Water vapor permeability (WVP) was determined because this property will serve as an indicator to analyze the stability of the food inside the package. Given the above, the permeability measurement was performed on the films prepared with pure SiO_2_ and with the particles obtained by methods M1 and M2. The results are summarized in Table 4; the pure PLA film presented the lowest WVP of 3.45 × 10^−8^ g Pa^−1^ h^−1^ m^−2^ regarding all films. The PLA film with pure SiO_2_ was the one that presented the highest permeability (8.45 × 10^−8^ g Pa^−1^ h^−1^ m^−2^), this could be due to the porosity of pure SiO_2_ that produced an increase in spaces in the polymeric matrix, and this facilitated the transfer of water vapor between the stored food and the external medium. Films prepared with nanoparticles from methods M1 and M2 presented a WVP of 7.93 × 10^−8^ g Pa^−1^ h^−1^ m^−2^ and 5.50 × 10^−8^ g Pa^−1^ h^−1^ m^−2^, respectively. With the above information, it can be said that the films are semi-permeable, which could be interpreted as beneficial for use in fresh foods that need to maintain a certain percentage of moisture to preserve their nutritional properties [90].

Several studies report increases and decreases in WVP, such as the one performed by Qin et al. [27]; they elaborated PLA films with bergamot (BO), lemongrass (LO), rosemary (RO), and clove (CO) essential oil at 9% wt, and reported that the films containing the essential oils increased the WVP of pure PLA from 1.96 × 10^−14^ kg m m^−2^ s^−1^ Pa^−1^ to 2.03 × 10^−14^ kg m m^−2^ s^−1^ Pa^−1^, (BO), 1.69 × 10^−14^ kg m^−2^ s^−1^ Pa^−1^, (LE), 1.54 × 10^−14^ kg m m^−2^ s^−1^ Pa^−1^, (RO) and 1. × 10^−14^ kg m^−2^ s^−1^ Pa^−1^ (CO). Javidit et al. [25] prepared PLA films with oregano essential oil at 1 and 1.5 wt%, finding that the water vapor permeability was reduced for pure PLA from 1.89 × 10^−8^ kg m m^−2^ s^−1^ Pa^−1^ to 1.25 89 × 10^−8^ kg m^−2^ s^−1^ Pa^−1^ at 1 wt% concentration, from 1.7289 × 10^−8^ kg m^−2^ s^−1^ Pa^−1^ at 1.5%wt concentration. Yu et al. [55] prepared PLA films reinforced with acetyl cellulose and ZnO nanoparticles and found that the permeability was reduced in higher proportion when nanoparticles were added at 3% concentration from 1.14 × 10^−11^ g m^−2^ Pa^−1^ s^−1^ of pure PLA to 0.73 × 10^−11^ g m^−2^ Pa^−1^ s^−1^. Finally, Praseptiangga et al. [54] prepared carrageenan films reinforced with SiO_2_ and ZnO nanoparticles, and the WVP of the film was slightly reduced when SiO_2_ nanoparticles were added, from 1.059 to 1.035 × 10^−6^ g h^−1^ m Pa^−1^; however, when combined at 1:1 SiO_2_/ZnO ratio an improvement was found to 1.011 × 10^−11^ g m^−2^ Pa^−1^ s^−1^. Based on the previous studies, the WVP in films depends on intrinsic factors such as the sorption of the molecules present in the matrix as well as their diffusion and the crystallinity of the polymer.

#### 3.3.3. Water Contact Angle (WCA)

**Figure 11:**
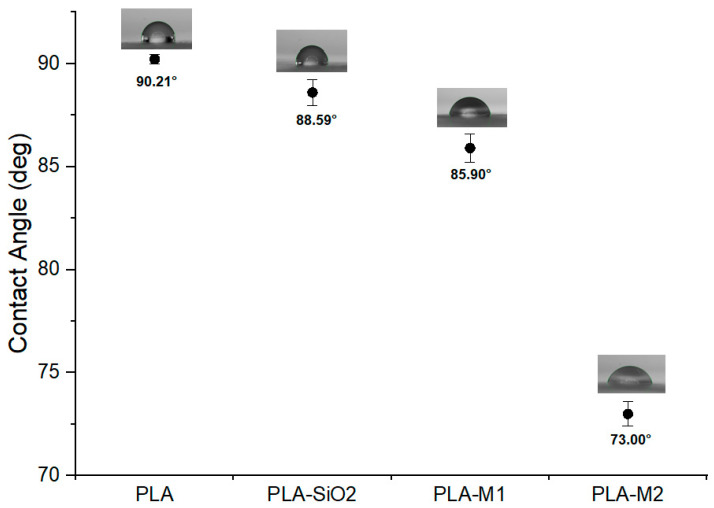
Contact angle of films PLA, PLA-SiO_2_, PLA-M1, and PLA-M2.

The contact angle indicates the affinity of a surface in contact with a liquid, in this case, water, and is also known as wettability. This property was determined on the films to know the effect of pure and CEO-loaded SiO_2_ nanoparticles on the wettability property. It is possible to observe in Figure 11 that the pure PLA film had a higher contact angle of 90.21° compared to the other films. The film containing the pure SiO_2_ nanoparticles and those modified with the M1 and M2 method significantly modified the contact angle (*p* < 0.05), resulting in values of 88.59°, 85.90°, and 73°, respectively. These values were less than 90°, indicating that the surface of these films is wettable and hydrophilic, so there is good adhesion between the film surface and the aqueous medium, in addition to a high surface free energy. The decrease in contact angle could be generated by pure SiO_2_ nanoparticles and not by the addition of CEO since previous studies, such as Mengting et al. [91], observed that due to a large number of hydroxyl groups and the high hydrophilicity of SiO_2_ nanoparticles considerably reduced this property, from 66° to 3° for their chitosan films with sodium phytate. However, Heydari et al. [19,92] mentioned that this property was increased in PLA films containing pure ZnO nanoparticles and peppermint essential oil and multiflora, corroborating that SiO_2_ nanoparticles in the polymer matrix generate hydrophilic surfaces even when CEO is included. Other studies have reported lower contact angle values, such as Roy et al. [9], who developed PLA films with curcumin as the active component and reported a contact angle for pure PLA of 71.2° while they found a slight increase of 74.3° when curcumin was added at a concentration of 1.5 wt%, indicating that the films had a hydrophobic character. Javidi et al. [25] evaluated PLA films with Origanum vulgare essential oil and reported that the contact angle was higher with increasing oil concentration; pure PLA presented a value of 61.20° and changed to 94.03° with the essential oil at a concentration of 1.5 wt%. Ranjbar et al. [93] prepared PLA/keratin nanofibers reinforced with nanofibrillated natural chitosan and ZnO nanoparticles. The combination of these materials showed that the contact angle decreased from 56° to 41°when the nanoparticles were added. The interaction of the components can help to increase or decrease this property so they do not follow a specific behavior.

#### 3.3.4. Mechanical Testing

Mechanical properties are very important parameters to consider when developing active packaging, as they must have good resistance to damage during handling and transportation to ensure that the product remains in good condition [25]. Table 4 shows the results of the mechanical properties, tensile strength (TS), Young’s modulus (YM), and elongation at break (EB) of pure PLA films and nanoparticles modified with the M1 and M2 methods. It can be observed that the TS of pure PLA was 27.06 MPa, and the film with pure SiO_2_ increased this value by 2.76% since it presented a TS of 28.06 MPa, which is attributed to the hydrogen bonding between the SiO_2_ nanoparticles and the hydroxyl groups of the polymeric matrix. This behavior was also observed by Singh et al. [94], Palma et al. [36], Casalini et al. [95], Lukic et al. [72], and Liu et al. [96]. However, the films containing CEO decreased the TS by 7.9% and 3.97%, having a minimum value of 25.13 MPa for the PLA-M2 system, generating a more brittle and easily broken film when subjected to a force. This effect could be attributed to the interaction of the CEO in the film network, indicating that discontinuous spaces are being generated, and, therefore, there is a disorder of the polymeric chains, which in turn causes a decrease in stiffness and strength [97]. This effect was observed by Rungsima et al. [98], Radusin et al. [8], and Heydari et al. [92], in which the oil concentration is increased, and hence these properties are reduced.

On the other hand, when evaluating the EB, a low value of 5.08% was obtained for the pure PLA film, indicating that this polymer is not easy to manipulate. In comparison, the films developed with SiO_2_ showed an increase of 12.3%. However, the films with particles modified with CEO showed a considerable increase; PLA-M2 had 63.77%, while the PLA-M1 system had a more significant increase since its increase was double since the essential oils interacting with the polymers act as plasticizers. This effect has been observed in several studies, where the addition of essential oil increased the spaces in the polymeric matrix, thus reducing the intermolecular forces, and therefore these can move easily without breaking; this property is acceptable for the manufacture of food packaging [8,99,100]. Martins et al. [101] observed a slight increase in elongation when green tea essential acid was added to PLA films, from 3.63 to 4.94%. Celebi et al. [26] observed that %EB increased twofold when thymol essential oil and carvacrol were added from 2.9 to 6.8%. Villegas et al. [102] similarly observed this increase in PLA films with cinnamon essential oil and thymol. A greater increase was observed when thymol was added, increasing the %EB from 10 to 120%.

As expected, Young’s modulus was significantly reduced by 45.98%, especially for the films prepared with the M1 and M2 method. The above indicates that the films can easily undergo deformation when an external force is applied, which is a similar characteristic to those present in food packaging bags. It is possible to notice that the PLA-SiO_2_ system increased Young’s modulus by 15.38%; this could be attributed to the fact that SiO_2_ is functioning as a nanofiller in the empty spaces of the polymeric network generating strong interactions that prevent the chains from moving easily and therefore reduce their flexibility [86,103,104,105]. A higher result than that obtained has been reported by Brancewicz et al. [106] of 2.344 Mpa for pure PLA. Torres et al. [107] reported that PLA in fiber form with essential oil with Propolis powder extract Young’s modulus decreased as the extract concentration increased from 36.6 MPa to 7 MPa. Torres et al. [107] report that when they added thymol essential oil to PLA films, Young’s modulus was considerably reduced from 1215 to 45.10 MPa.

#### 3.3.5. DSC

**Figure 12:**
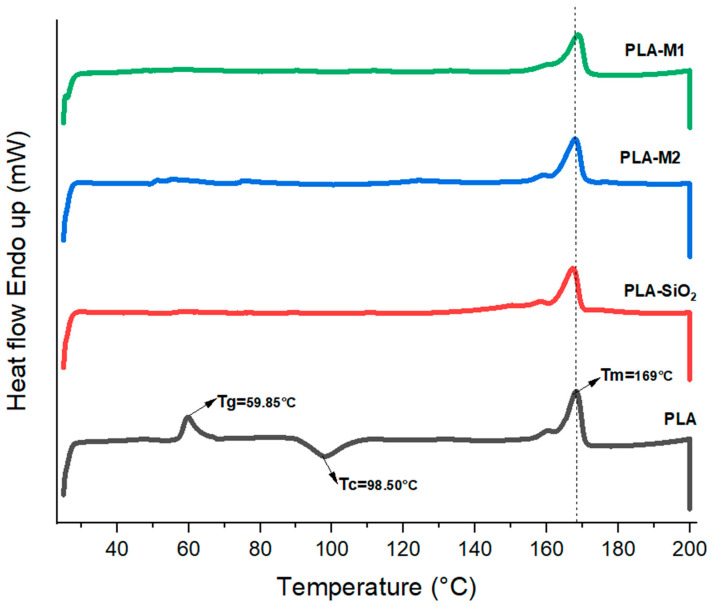
Thermogram of PLA and composites obtained by DSC. Vertical line indicates the melting temperature of neat PLA.

In order to know the thermal transitions of the formulated films, thermograms were obtained by differential scanning calorimetry (DSC). The melting temperature (Tm), crystallization temperature (Tc), and glass transition temperature (Tg) are summarized in Table 4; in addition, these data were plotted in Figure 12. On the one hand, PLA presented a Tg of 59.95 °C, but in the other films prepared with SiO_2_ (pristine and modified), this transition was not observed, which could be because the nanoparticles reduced the free volume of the polymeric chains, which prevents their movement at temperatures below 100 °C. The crystallization temperature of pure PLA was 98.50 °C, characterized by an exothermic peak, indicating a slow crystallization during the heating, leading to the reorganization of the polymeric chains and their crystallization temperature before reaching their melting temperature, which was 169 °C. This behavior and similar results have been observed in other research works [46,64,77].

On the other hand, it can be noticed that the films that had SiO_2_, SiO_2_-M1, and SiO_2_-M2 nanoparticles lacked a crystallization temperature during the heating. This could be due to the fact that the nanoparticles immersed in the polymeric matrix contained CEO and were prepared with chloroform. Therefore, in this analysis, if we detect the crystalline structure of the material prior to cold crystallization, then the crystallization exotherm only reflects what crystallized during film formation by the casting method. The exotherm showed that the melting temperature was similar to that of pure PLA between 166–169 °C. Table 4 also shows the melting enthalpy $\left(\Delta H_{m}\right)$, crystallization enthalpy $\left(\Delta H_{c}\right)$, and the degree of crystallization (Xc) obtained from the DSC curve. These results indicate that PLA presented a very low crystallization degree of 10.43%, while the highest degree of crystallization was reached when SiO_2_ nanoparticles (43.03%) were added. However, when SiO_2_-M1 and SiO_2_-M2 nanoparticles were added, these reduced the crystallinity, obtaining 26.25 and 35.89%, respectively.

Some works mention that metal oxide nanoparticles have improved the crystallinity of PLA, as well as its Tg. The study conducted by Zhang et al. [108] mentioned that adding ZnO nanoparticles at 0.25% increased the Tg of pure PLA from 59.04 °C to 63.02 °C, as well as the degree of crystallization from 4.22% to 12.95%. Łopusiewicz et al. [109] made PLA films with fungal melanin as an active component and reported a Tg of 61 °C with a lower Tm than that obtained in the present work of 148 °C and with an increase in the crystallinity from 0.23% to 1.61% with 0.2% of fungal melanin. Cvek et al. [110] elaborated on PLA and propylene carbonate composites loaded with curcumin. When evaluating their effects on thermal properties, they observed that the addition of curcumin did not significantly alter their transition temperatures, Tg (56–59 °C), the Tc (125.5–127.4 °C) and Tm (148.5–148.8 °C); however, the degree of crystallization had a decrease from 1.1 to 0.2%, due to curcumin acts as a plasticizer and therefore reduces the crystallinity in the films; this same effect is observed when essential oils were added.

### 3.4. Storage Analysis of Apple Samples

#### 3.4.1. Weight Loss

**Figure 13:**
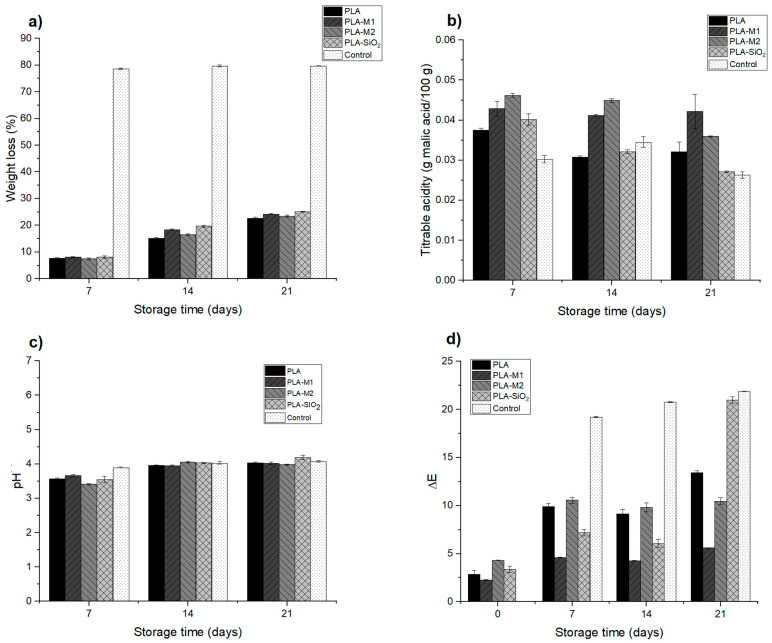
Physico-chemical evaluation of apple storage: (**a**) weight loss, (**b**) titratable acidity, (**c**) pH, and (**d**) color difference.

The weight loss of food is influenced by the type of material and storage conditions, as well as by the respiratory rate of some foods. Generally, the packages that maintain weight loss are those in which permeability is minimal since it reduces weight loss in the form of water vapor [111]. A conventional package that has good barrier properties is high-density polyethylene or polypropylene, which has been used in various presentations and has good resistance to shock and impact. This type of material is used to store vegetables, fruits, cereals, nuts, and seeds for more than 21 days. The weight loss in the apple pieces was determined to evaluate the films as suitable packaging to be used in food since their main function is to protect the food from external factors and maintain its properties, in this case, humidity and avoiding water loss in the form of weight [35]. Table 5 shows the final results of storage at 21 days; it can be noticed that there is a large significant weight loss (*p* < 0.05) for the control sample, which was not stored in packaging; this indicates that it lost a considerable amount of water by 78% compared to the samples stored in the package, which only had a loss of 22.71%, 24.24%, 25.05%, and 23.43% for PLA, PLA-M1, PLA-SiO_2,_ and PLA-M2 systems respectively. Figure 13a shows the evolution of weight loss during storage, observing that this variable increased as the days passed; however, the other samples placed inside the active container maintained a greater amount of water and freshness with a loss of less than 25%. A similar effect was observed in the study by Chen et al. [112] when storing apple slices in films composed of sodium alginate and thymol essential oil, as well as in the study by Sganzerla et al. [113], who elaborated on citrus starch and pectin films for apple storage. The results show that the packaging fulfills its function of protecting the product and, therefore, avoids a greater loss of water by transpiration of the apple during storage, thus prolonging its shelf life.

#### 3.4.2. Titratable Acidity and pH

Titratable acidity (TA) is an important quality parameter in foods, especially fruits because they are rich in organic acids. During the storage process, a decrease in this parameter was observed, which could be due to the degradation of organic acids inside the fruit caused by metabolic reactions that trigger modifications in flavor, color, and stability [114,115,116]. Figure 13b and Table 5 show the results at the end of storage. It can be observed that the evolution of the titratable acidity in the control sample decreased considerably. In contrast, the apple samples stored in the packaging after 21 days of storage maintained a TA very close to the initial sample, which was 0.0502; there was only a decrease of 16% (0.042) and 30% (0.035) for the samples in PLA-M1 and PLA-M2 packaging, respectively.

Also, it can be observed that the PLA-SiO_2_ sample presented a significant decrease of 46% (0.027), very close to that obtained in the control sample that presented a decrease of 48% (0.026), which, as expected, would suffer a greater degradation and loss of nutrients when exposed to the elements. In the previous section, the PLA-SiO_2_ system presented a high permeability value to water vapor; possibly, this generates a greater transfer of oxygen and components that accelerate the metabolism of organic acids and significantly affect the quality of the apples. PLA-M1 and PLA-M2 packaging slowed down the degradation of malic acid in apples, which could be attributed to the antioxidant properties of CEO. This behavior was observed in the study of Lan et al. [117], who formulated polyvinyl alcohol composite films with polyphenol Tea for strawberry storage. The same occurred in the study of Zhang et al. [86], who developed chitosan films for apple storage.

As for the evaluation of pH, it was determined during the storage of the apple. Each sample was measured during the 21 days, as shown in Figure 13c. Initially, the control sample presented a pH value of 3.09; as the days passed, a slight increase in pH of 4.08 was observed at the storage end; the results are summarized in Table 5. As for the samples deposited in the developed containers, it can be observed that they followed the same behavior with slight increases, for the sample stored in the PLA-M1 system after 7 days presented a pH of 3.66 and at the end of 4.02; likewise, for the PLA-M2 film the sample presented a pH of 3.41 and at the end of 3.98; finally, the sample stored in the PLA-SiO_2_ film had a significant increase in pH from 3.55 to 4.19. This could be due to the biological processes of metabolism in apples, as the degradation of organic acids and sugars causes effects on pH [118]. This behavior could be related to the TA result obtained in the sample stored in this film.

#### 3.4.3. Color

The measurement of color is another important parameter in food since this property generates the acceptance or rejection of consuming a product. In some foods, the enzyme polyphenoloxidase (PPO) has a visible growth in the so-called enzymatic browning, where fruits that have this enzyme begin to present dark brown spots when they come into contact with air; for example, avocado, banana, pear, and apples to mention a few. In quantitative terms, it is possible to know the shades of the apple samples through three magnitudes (brightness, hue, and saturation) and observe the color difference to find out the effects caused by the enzyme polyphenol oxidase in contact with the air, which causes enzymatic browning.

**Figure 14:**
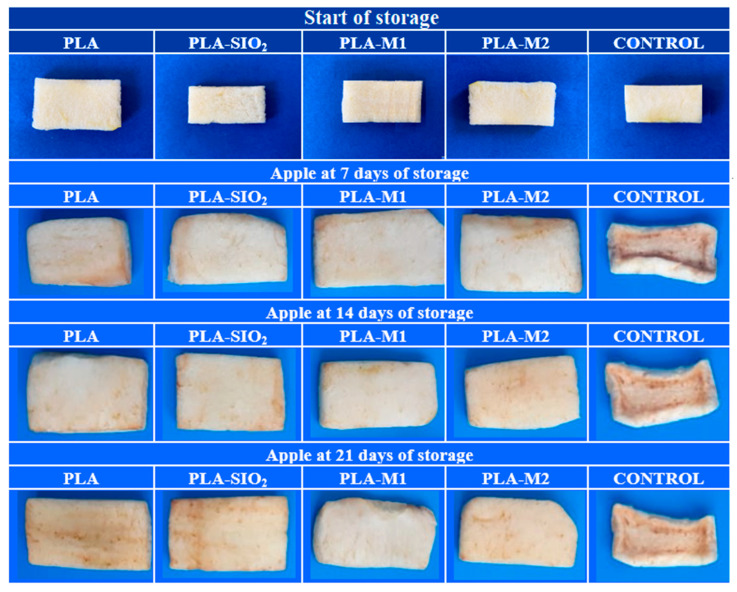
Visual appearance of apples stored for 21 days.

Table 4 summarizes the values obtained for the color difference, which is the greatest interest parameter at the storage end. It is possible to observe that the sample contained in the PLA-SiO_2_ film had a significant color difference (*p* < 0.05) of 20.96, very close to the value obtained by the control sample of 21.89, this could be referred that the film did not function as a good barrier between the sample and the outside, which caused a greater oxygen transfer that favored the growth of the polyphenol oxidase enzyme, in addition to the fact that pure SiO_2_ does not have a good antioxidant capacity to inhibit the growth of the enzyme. In contrast, the samples stored in PLA-M1 and PLA-M2 films had a color difference of 5.59 and 10.47, respectively, these results are very favorable for the preservation of apples, so it is possible to ensure that the essential oil of cinnamon helps to inhibit the growth of the enzyme due to its antioxidant properties that were captured in the SiO_2_ nanoparticles and maintained in the PLA matrix. In Figure 13d, it is possible to appreciate these results graphically and visually in the photos of Figure 14, where it can be seen the texture and appearance of the samples.

## 4. Conclusions

In this study, SiO_2_ nanoparticles were chemically modified with cinnamon essential oil by applying two methods (M1 and M2) to achieve antioxidant and antimicrobial properties. This was confirmed by FTIR and TGA analysis, which showed the presence of the cinnamaldehyde group, typical of cinnamon essential oil. The modified nanoparticles were found to be non-toxic and to have the ability to inhibit the growth of Gram-positive and Gram-negative bacteria, making them suitable for food applications. With the modified nanoparticles, PLA films were developed, and significant changes were observed; the films became more flexible when SiO_2_-M1 nanoparticles were added, causing the material to increase its elongation by 50%; this led to low tensile strength and Young’s modulus. The same occurred when SiO_2_-M2 nanoparticles were added. However, when pure SiO_2_ nanoparticles were added, the film became more rigid and difficult to handle, demonstrating that cinnamon essential oil functions as a plasticizer. The addition of SiO_2_, SiO_2_-M1, and SiO_2_-M2 nanoparticles decreased the percentage of water solubility of the films, so they may be less susceptible to degradation. In addition, they did not affect the molecular structure of PLA and slightly reduced the percentage of crystallinity of the polymer. As for permeability, slight increases were obtained, making the films more porous, which facilitated the diffusion of water vapor and the wettability of the films, generating contact angles of less than 90°. The PLA- SiO_2_ film was the most permeable (8.45 × 10–8 g Pa^−1^ h^−1^ m^−2^), which influenced the preservation of the apples by accelerating their deterioration. The SiO_2_-M1 and SiO_2_-M2 nanoparticles transferred antioxidant properties to the films, which was useful for the preservation of apples since they maintained a lower loss of weight, color, titratable acidity, and pH during 21 days of storage, so these materials could be used for the preparation of active packaging.

## Figures and Tables

**Table 1:** Results of the antimicrobial activity expressed as the minimum inhibitory concentration in nanoparticles of SiO_2_ pure, SiO_2_-M1 and SiO_2_-M2.

Sample	*E. coli* (MIC)	*S. aureus* (MIC)
SiO_2_-M1	45 µg mL^−1^	90 µg mL^−1^
SiO_2_-M2	11 µg mL^−1^	750 µg mL^−1^
SiO_2_	-	-

-: Not detected.

**Table 2:** Results of the antimicrobial activity expressed as % inhibition in the PLA films.

Sample	*E. coli* (% Inhibition)	*S. aureus* (% Inhibition)
PLA	0.43	0.92
PLA-M1	3.54	2.59
PLA-M2	3.44	1.29
PLA-SiO_2_	0.96	0.92

**Table 3:** Water solubility, water vapor permeability, tensile strength, elongation at break, and Young’s module of systems: PLA pure, PLA-SiO_2_, PLA-M1, and PLA-M2.

Sample	WS (%) ±SD	WVP (g Pa^−1^ h^−1^ m^−2^) ± SD	TS (MPa) ± SD	EB (%) ± SD	YM (MPa) ± SD
PLA	15.49 ± 0.213 ^a^	3.45 × 10^−8^ ± 0.721 ^a^	27.06 ± 2.57 ^ab^	5.08 ± 1.64	0.324 ± 0.183 ^b^
PLA-SiO_2_	7.24 ± 0.106 ^a^	8.45 × 10^−8^ ± 0.002 ^a^	28.06 ± 3.08 ^b^	4.44 ± 2.55	0.368 ± 0.048 ^a^
PLA-M1	6.41 ± 0.359 ^a^	7.93 × 10^−8^ ± 0.052 ^a^	26.22 ± 1.55 ^a^	10.32 ± 5.98	0.175 ± 0.092 ^a^
PLA-M2	6.37 ± 0.472 ^a^	9.50 × 10^−8^ ± 0.020 ^a^	25.13 ± 2.80 ^c^	8.32 ± 3.56	0.175 ± 0.094 ^a^

Values are expressed as mean ± standard deviation. Different letters in the same column indicate significant differences (*p* < 0.05).

**Table 4:** Results of thermal transitions of PLA and its composites as determined by DSC.

Sample	Glass Transition	Cold Crystallization		Melting		Total Crystallinity
	$T_{g}\;\;\left({}^{\circ}C\right)$	$T_{c}\left({}^{\circ}C\right)$	$\Delta H_{c}$	$T_{m}\left({}^{\circ}C\right)$	$\Delta H_{m}$	$X_{c}$
PLA	59.95	98.50	18.68	168.62	28.38	10.43
PLA-SiO_2_	-	-	-	167.68	40.02	43.03
PLA-M1	-	-	-	167.92	24.42	26.25
PLA-M2	-	-	-	169	33.38	35.89

**Table 5:** Weight loss, titratable acidity, color difference, and pH in the samples stored in the active packages at the end of the 21-day storage period.

Sample	Weight Loss (%)	Titratable Acidity	pH	Color Difference ΔE
PLA	22.71 ± 0.376 ^a^	0.032 ± 0.002 ^a^	4.03 ± 0.002 ^a^	13.40 ± 0.034 ^ab^
PLA-SiO_2_	25.05 ± 0.280 ^a^	0.027 ± 0.004 ^a^	4.19 ± 0.002 ^a^	20.96 ± 0.053 ^a^
PLA-M1	24.24 ± 0.399 ^ab^	0.042 ± 0.004 ^a^	4.02 ± 0.002 ^a^	5.59 ± 0.034 ^ab^
PLA-M2 CONTROL	23.43 ± 0.353 ^b^	0.035 ± 0.003 ^a^	3.98 ± 0.002 ^a^	10.47 ± 0.043 ^a^
	79.78 ± 0.178 ^a^	0.026 ± 0.008 ^a^	4.08 ± 0.002 ^a^	21.89 ± 0.047 ^a^

Values are expressed as mean ± standard deviation. Different letters in the same column indicate significant differences (*p <* 0.05).

## Data Availability

Data is contained within the article.
